# Supplementary material for: Socio-demographic factors associated with early antenatal care visits among pregnant women in Malawi: 2004–2016
Source: PLoS One. 2022 Feb 8;17(2):e0263650. doi: 10.1371/journal.pone.0263650 (PMC8824333; doi:10.1371/journal.pone.0263650)
Supplement: Attachment — Submitted filename: Response to Reviewers.docx [file pone.0263650.s003.docx]

**Response to Editor Comments**

1.) **“The authors seem to have misunderstood the message about the weights. The current description of the weights use is for just a single survey procedure. When there is a combination of surveys the weights must be corrected. Please do see the comments from the reviewer bellow. And please add the details of such weighting procedure in the statistical analysis section.”**

Thank you for highlighting this. We have amended the weighting procedure as directed and made changes to the manuscript accordingly. Please see response to reviewer three for a detailed description of our actions.

2.) **“Put the ORs and 95%CI for the factors you mention in the results”**

Thank you, we have now added the ORs and 95% CI for all results that are reported in the abstract. Changes can be seen at lines 34-44, as indicated by highlighting. In addition, we have added that the results for the primary outcome into the abstract as well as seen at line 35.

3.) “**There still “STATA” instead of “Stata” in the abstract.”**

Thank you for highlighting. This has been amended as show at line 25.

4.) “**Line 91 “..” correct to be “.”**”

Thank you for highlighting. This has been amended as show at line 99.

5.) **“Methods - There is nowhere in this manuscript a description of what women are included in the analysis. Line 105 says it is a secondary analysis of the women’s questionnaire data. Not many readers of PlosONE will know what is this questionnaire. Please state briefly what women are included in the survey (women with a pregnancy in the last 5 years prior to the survey, for example). Such statement should be added to the abstract as well.”**

We have now amended the manuscript to include a description of the survey sample in both the abstract, at line 25-27, and in the main text at line 115 - 122.

6.) **“Results - Table 1 and 2 are using weights? Please add footnote explaining that.”**

Thank you for highlighting. We have now added footnotes to all tables and figures to show that weights were used in calculating proportions/percentages and odds ratios.

**Response to Reviewer Comments**

**a) On the question of the number of women having a most recent birth (in the 24 months before the survey); in my previous comments, I was pointing to having a column indicating the overall number of women aged 15-49 interviewed for the main survey vs the women having a most recent birth (in the 24 months before the survey). For example, in there a total of 11,698 women (15-49) were interviewed for the main questionnaire vs 6012 (51%%). Also, the captions of Tables 1 and 2 may be misleading at first sight because it talks of women interviewed, rather than women with a recent birth (24 months before the survey). Thus, if one puts a column on Table 1 indicating the number of women from which this sample came, e.g. 2,407 women in the age group 15-19 vs 602 analyzed here, etc.**

Thank you for the clarification of your previous comment. We feel that addition of the column may not be of any value as such women to be included in the other column did not have a recent birth of whom their antenatal care is being assessed in terms of healthcare seeking behaviour.

Additionally, we have updated the titles of table 1 and 2 to more accurately reflect their contents, in this case that they refer exclusively to women who delivered in the past five years of the survey.

b) **I applaud that the authors in accounting for the design of the surveys by way of weighted analyses. And in most surveys, designs weights are combined with missing data and non-response. However, when analyzing combined data of multiple MDHS datasets, individual survey weights would need to be adjusted correctly to represent a case in the combined data sets. See for example.**

Thank you very much for the direction regarding the survey weights and for providing additional sources that we were able to use to strengthen our analysis. We have now employed an equal weights approach as heighted in Friedman et al (2002). We have provided a description of our methods at line 149 and added Friedman et al’s paper to the reference list at number 25.

Following this weight approach we found that ‘frequency of watching television’ which was significant in the previous analysis is no longer significant factor in the presence of other variables. Further, different percentages as shown by Tables and Figures also changed. Please refer to the Tables and Figures for more details on the change.

**c) On the question of 8+ ANC visits, one could use it as sensitivity analysis.**

As for 8+ ANC, we had 513 (2% of 26,234) women who had at least 8 ANC visits. We did not perform the sensitivity analysis since the numbers are small and a future analysis should examine this after when the recommendations have been in place for longer.
